# Modeling and Evaluation of Piezoelectric Transducer (PZT)-Based Through-Metal Energy and Data Transfer †

**DOI:** 10.3390/s20113304

**Published:** 2020-06-10

**Authors:** Lianghui Ding, Kehong Chen, Falong Huang, Feng Yang, Liang Qian

**Affiliations:** Department of Electronic Engineering, Shanghai Jiao Tong University, Shanghai 200240, China; khchen@sjtu.edu.cn (K.C.); huangfalong@alumni.sjtu.edu.cn (F.H.); yangfeng@sjtu.edu.cn (F.Y.); lqian@sjtu.edu.cn (L.Q.)

**Keywords:** piezoelectric transducer, spatial equivalent plane acoustic pressure, through-metal power transfer, through-metal communication

## Abstract

Through-metal transfer of energy and data using piezoelectric transduce can avoid the potential leakage problem caused by physical penetrations and wired feed-through. The through-metal transfer efficiency of energy or data is determined by the relative pressure on the receiving PZT (piezoelectric transducer). Hence, in this paper, we first propose the Spatial Equivalent Plane Acoustic Pressure (SEPAP), which is defined as the integration of the acoustic pressure over the receiving area, to model the pressure on the receiving PZT. Then we analyze the features of SEPAP and the factors impacting it by utilizing COMSOL. Furthermore, we propose a low-cost and small-size prototype for simultaneous transfer of energy and bidirectional communication through metal by using two pairs of PZTs working on different resonant frequencies. Extensive experiment has been done on evaluating the match between SEPAP transfer efficiency and the power transfer efficiency and analyzing the achievable data rate for bi-directional communication. Test through a 20 mm aluminum alloy plate shows that power transmission with efficiency 20.3% and data communication rate up to 38.4 Kbps can be achieved simultaneously.

## 1. Introduction

In order to improve the structural strength and anti-corrosion performance, metal materials are the first choice for underwater devices, aerospace equipment and radiation isolation facilities. However, power supply, control and communication between devices located on the isolated sides of metal barriers is always a challenging task taking into account the strength requirement. Traditionally, physical penetrations and wired feed-through have been the mainstream because wireless coupling is limited by the metal. However, physical penetrations inevitably impact the structural integrity and impermeability of metal shells. Therefore, penetration-free techniques for transmitting data and power through metal barriers is demanded [1,2,3].

**Figure 1:**
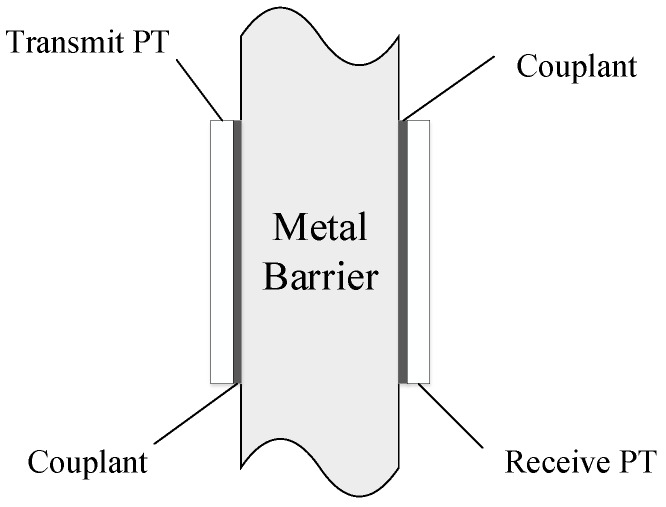
Typical acoustic-electric system.

As elastic waves can easily propagate through metal barriers, it has attracted wide attention from academia and industry. The piezoelectric materials can be used for bidirectional conversion between electrical signals and mechanical waves (ultrasonic waves) using piezoelectric effect and inverse piezoelectric effect. A typical acoustic-electric system called sandwiched plate PZT (piezoelectric transducer) is shown in Figure 1 [4]. At the transmitter (TX) side, modulated electrical signal is converted into elastic wave by a PZT, and the elastic wave is propagated through the couplant into the metal. In the receiver (RX) side, PZT collect the elastic wave via the couplant and converts it to the electrical signal.

Through-metal transmission systems based on PZT has been proven to be feasible. However, it is challenging to establish the power transfer model since the system generally works in the acoustic near-field region for high efficiency. Two main methods have been employed in previous literature on piezoelectric power transfer through metal: practical experiment [5,6,7,8,9,10,11] and simulation analysis [12,13,14,15,16,17,18,19].

By using practical experiment, S. Sherrit et al. studied three different coupling methods between the transducer and the metal barrier, including bolting, mechanical clamping and epoxy bonding [5]. Results show that the epoxy resin coupling achieves the best balance between the power transmission efficiency and the structural complexity. X. Bao et al. verified that transducer coupled with epoxy can penetrate 3.4 mm metal barrier to achieve 88% power efficiency [6,7]. It was shown in [8] that up to 50 W power can penetrate a 2.5-inch steel barrier. Through-metal power transfer systems with low complexity and small size for practical application have been proposed in [9,10,11].

In simulation analysis, S. Roa-Prada et al. established an acoustic field model of piston transducer in the barrier, and analyzed the axial response of the transducer in near region and the influence of beam spreading on power losses [13,14]. K. R. Wilt et al. proposed an axisymmetric finite element model and described the influence of mechanical parameters on power transmission efficiency, including metal material, thickness, the couplant and the transducer size [15]. Besides, the distribution of acoustic pressure in metal barriers is simulated by COMSOL in [16]. They also proposed ABCD parameters based on sandwiched plate PT system [17], then derived a decoupled layer model considering forward and reverse traveling pressure waves [18]. Z. Chang find that the loss of Lamb wave (i.e., plate wave) is an important factor in the loss of sound power [19].

However, to our best knowledge, the relationship between the acoustic pressure and the power transfer efficiency is still an open question, because the acoustic pressure distribution in the near field composed of lots of transmission sources is very complicated.

In a lot of practical applications, simultaneous transfer of power and data through metal are necessary. Typical solutions with single pair and two pairs of coaxially aligned PZT have been reported in [8,20,21,22,23] and [10,24,25] respectively. In solutions with single-pair PZT [8,20,21,22,23], low-rate amplitude modulation has been used for communications from the “outside” to “inside” together with power transfer, while changing of the electrical load on the terminals of the inside PZT has been used for communication from “inside” to “outside”. Single-pair PZT solution can minimizes the size, while the communication from “outside” to “inside” is pretty low. In the solution with two pairs of PZTs coupling on both sides of a steel plate, Lawry, T.J. et al. achieves the data rate up to 12.4 Mbps by using OFDM modulation [10]. However, the complexity and the cost is very high. Therefore, in this paper, we will propose a low-cost and small-sized prototype by using two-pairs of PZTs for simultaneous through-metal power and data transfer.

Since the transferred power through metal is determined by the pressure on the receiving PZT, we first propose model it as Spatial Equivalent Plane Acoustic Pressure (SEPAP), which is defined as the integration of the acoustic pressure over a given circular region. By using COMSOL simulation, the influence of the metal thickness and the transducer size on SEPAP is then studied.

Then, we propose a low-cost and small-sized prototype for simultaneous through-metal power and data transfer by using two pairs of PZTs working on different resonant frequencies. At the TX side of the power transfer system, we use a high-voltage operational amplifier to amplify the sinusoidal signal generated from a direct digital synthesizer (DDS) source. At the RX side, the power is output from the received AC signal sequentially through a full-bridge rectifier and a DC-DC regulator. In the communication system, we use Amplitude Shift Keying (ASK) with the rate matching the ISI-free link rate as the modulation scheme, and Time Division Duplex (TDD) as the duplex scheme.

Extensive experiment has been done to show that the SEPAP transfer efficiency can accurately characterize the power transfer efficiency regardless of the barrier thickness and the PZT size. According to the measured channel response, we analyzed the coherence bandwidth and the achievable data rate for communication. Practical test through a 20 mm aluminum alloy plate demonstrates that power transmission efficiency higher than 20% and data communication rate faster than 38.4 Kbps can be achieved simultaneously.

The remaining part of this paper is arranged as follows: We introduce the propagation characteristics of acoustic-electric channel of PZT-based sandwiched structure in Section 2. Then we model the equivalent pressure transferred through metal by SEPAP and analyze its feature by using COMSOL in Section 3. Furthermore, we present the design of the prototype for simultaneous transfer of power and data through metal in Section 4. After that, the experiment verification and analysis are given in Section 5, and the whole paper is concluded in Section 6.

## 2. System Model of Acoustic-Electric Channels

### 2.1. Acoustic-Electric Channel

**Figure 2:**
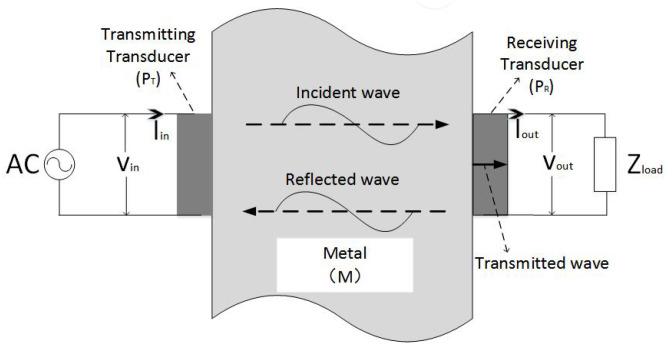
Electro-acoustic-electric channel structure.

We consider a system as shown in Figure 2. The transducer is coupled with epoxy resin on symmetrical sides of the metal barrier to form an electrical-acoustic-electrical channel. An Alternating Current (AC) source $V_{in}$ is loaded to the TX transducer ($P_{T}$), which converts the excitation signal to the mechanical wave through the inverse piezoelectric effect. The RX transducer ($P_{R}$) on the other side of the barrier converts the mechanical wave to the electric signal $V_{out}$ through the piezoelectric effect.

According to [26], we know that the plane wave propagating in the space medium satisfies the following equation

(1) $$p=\rho_{0}c_{0}u=Z_{0}u$$

where *p* is the spatial acoustic pressure, $\rho_{0}$ is the density of the medium, $c_{0}$ is the acoustic wave velocity in the medium, $Z_{0}$ is the acoustic characteristic impedance, and *u* is the particle velocity. At any position in the planar acoustic field, the acoustic pressure and the particle velocity have the same phase.

### 2.2. Propagation Model of PZT Channel

**Figure 3:**
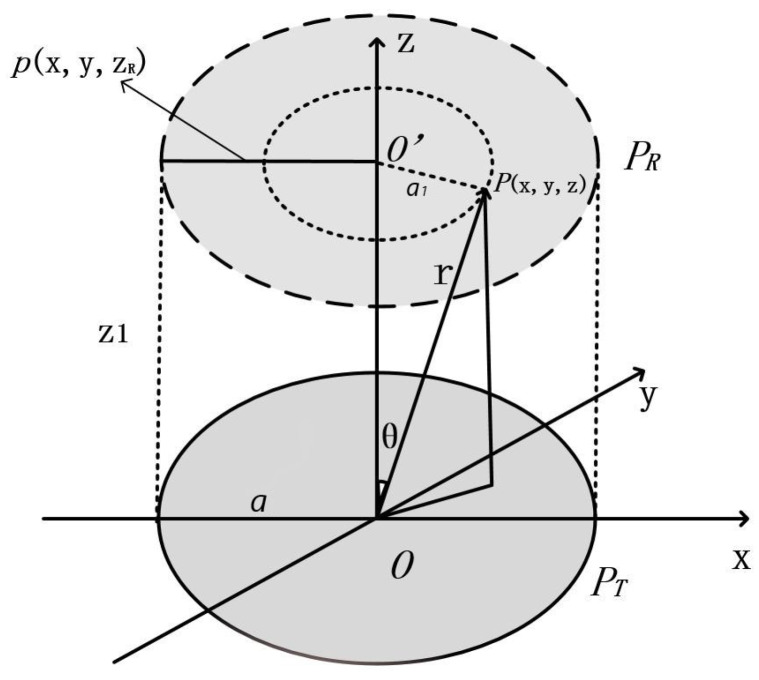
Piston acoustic source model.

According to acoustic-electric channel structure, we can model the TX transducer $P_{T}$ as a piston acoustic source formed by multiple point sources to simplify the analysis as shown in Figure 3.

The acoustic pressure at the point in the fluid medium (the metal medium can be approximated by this equation) with coordinate (x,y,z) in Figure 3 can be described by [27]

(2) $$p\left(x,y,z\right)=-i\omega \rho_{0}\;\int \int \;W\left(x^{\prime },y^{\prime }\right)\frac{exp(ikR\left(x^{\prime },y^{\prime }\right))}{4\pi R(x^{\prime },y^{\prime })}dx^{\prime }dy^{\prime }$$

where $W(x^{\prime },y^{\prime })$ is the acoustic source intensity per unit area, and *k* is the wave number, $\rho_{0}$ is the density of the medium in free space, *c* is the speed of sound waves in free space, and

(3) $$R\left(x^{\prime },y^{\prime }\right)=\sqrt{{\left(x-x^{\prime }\right)}^{2}+{\left(y-y^{\prime }\right)}^{2}+{\left(z-z^{\prime }\right)}^{2}}$$

(4) $$k=\frac{\omega }{c}=\frac{2\pi }{\lambda }$$

where ω and λ are the angular frequency and wavelength of the TX PZT respectively, *c* is the speed of light. The acoustic waves generated by transducer vibration can be divided into two regions: far-field and near-field. The boundary of two regions along the central axis is

(5) $$N=\frac{a^{2}}{\lambda }$$

where *a* is the radius of the transmitter PZT. The space of z>N belongs to the far-field, and space in z<N belongs to the near-field. In the far-field, the spatial acoustic pressure decreases with the increase of the distance from the source. However, in the near-field, the acoustic waves generated by the piston acoustic source interfere with each other; thus, the acoustic pressure in the near-field space varies with distance and location. It is noted from Equation (2) that the point acoustic pressure distribution in near-field space is complicated, and there is no general expression for the spatial plane acoustic pressure. However, for high penetration efficiency, through-metal power transfer generally works in the near-field.

## 3. Modeling on Efficiency of Through-Metal Power Transfer

### 3.1. Spatial Equivalent Acoustic Pressure (SEPAP)

As we know, the amount of power generated from a RX PZT is determined by the total pressure transferred from the TX PZT through the metal. We propose that we can use the surface integration of the pressure to indicate the power penetration efficiency.

Since $p(x,y,z_{R})$ is an instantaneous acoustic pressure, the value of which varies with time. Thus, we have to use the effective acoustic pressure to describe the steady-state feature. According to [27], the effective acoustic pressure is defined as the root mean square of acoustic pressure over a period.

(6) $$p_{e}\left(x,y,z_{R}\right)=\sqrt{\int_{0}^{T}p{\left(x,y,z_{R}\right)}^{2}dt}$$

Considering the model given in Section 2.2, for a plane $P_{R}$ with distance *R* from the transducer, we can use $p(x,y,z_{R})$ to denote the acoustic pressure function of the plane. According to Equations (2)–(4), we can find that the acoustic pressure of each point on a circular ring with radius $r=a_{1}$ in Figure 3 remains the same. Thus, the effective acoustic pressure $P_{e}\left(x,y,z_{R}\right)$ is also axial symmetric. We can define the Spatial Equivalent Acoustic Pressure (SEPAP) on the receiving PZT as

(7) $$p_{SEPAP}=\int_{0}^{b}2\pi bp_{e}\left(x,y,z_{R}\right)dx$$

where *b* is the radius of the RX PZT.

However, it is very hard to get the analytical solution of $p_{e}\left(x,y,z_{R}\right)$. Therefore, we propose that we can obtain $p_{e}\left(x,y,z_{R}\right)$ over a plane from the finite element tool, COMSOL, and use the surface integration as the Spatial Equivalent Acoustic Pressure (SEPAP).

### 3.2. Simulation Setup

We choose the transducer with the diameter of 50 mm and with the resonant frequency of 1 MHz. A circular aluminum plate with the diameter of 200 mm and the thickness of 150 mm is chosen as the barrier. To investigate the impact of transducer size on SEPAP, transducers with diameter of D=40 mm and D=25 mm have also been considered in Section 5.1. The material properties listed in Table 1 and the constitutive equation that piezoelectric materials follow [28,29] are imported into the COMSOL for simulation and analysis. The excitation voltage of the PZT is 10 $V_{PP}$.

### 3.3. Pressure Distribution on the Vertical Cross-Section of the Barrier

**Figure 4:**
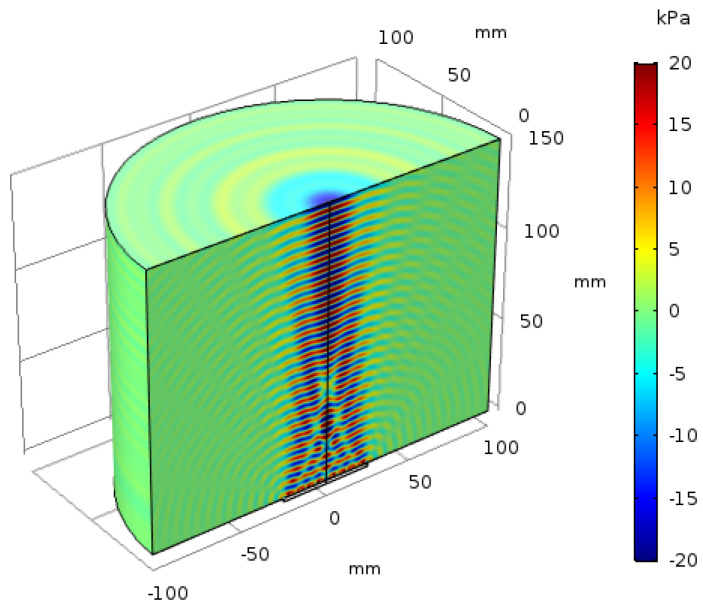
Acoustic pressure distribution on the vertical cross-section of the barrier.

The acoustic pressure distribution on the vertical cross-section of the barrier is shown in Figure 4. The maximum acoustic pressure in the section is 20 kPa. Counting the number of full waves in the acoustic beam (each peak is a full wave) indicates that the acoustic beam covers approximately 24 wavelengths. The acoustic pressure reaches its maximum value when the thickness of the metal barrier is an integral multiple of half acoustic wave length. In the far-field, the positive and negative acoustic pressure alternates and is very regular, whereas the acoustic pressure response in the near-field is highly irregular due to interference between acoustic waves.

**Figure 5:**
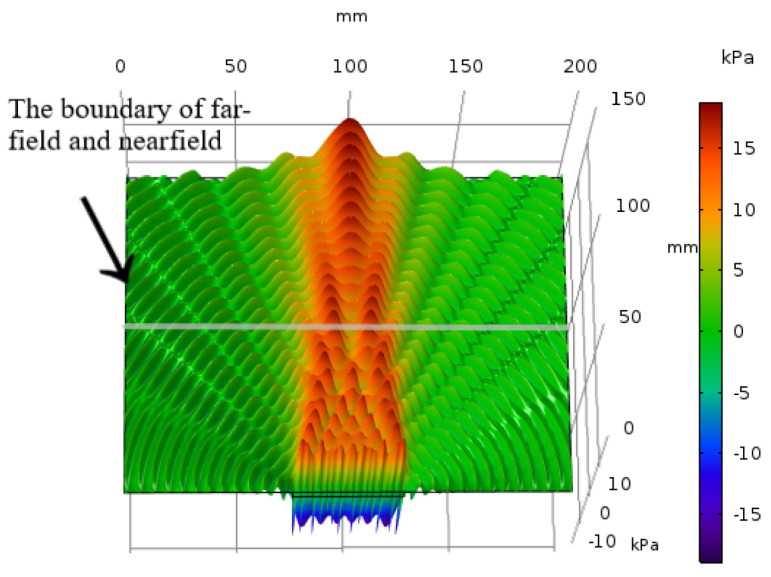
3-D acoustic pressure distribution on the vertical cross-section of the barrier.

The 3-D acoustic pressure distribution on the vertical cross-section of the barrier from the COMSOL simulation is shown in Figure 5. According to the wave distribution in Figure 5, we can find that the boundary between far-field and nearfield is about 100 mm. When the frequency of the stimulating signal is 1 MHz, we can also calculate the boundary of far-field and nearfield as $N_{D=50m}\approx 100$ mm according to 5. It proves that the simulation results from COMSOL simulation matches the theoretical acoustic analysis well. In the nearfield, the width of the acoustic wave radiation is equal to the transducer’s diameter, and the radiation range is approximately twice the diameter of the transducer. At the same time, the acoustic waves interfere with each other and the amplitude of the acoustic pressure fluctuates irregularly.

From Figure 5, it can be found that the acoustic power is concentrated on the main lobe, just a small amount of power leak from side lobes, and the number of side lobes which matches the result in [28] is D/λ=(50mm/6.4mm)≈7.

### 3.4. Pressure Distribution on the Horizontal Cross-Section of the Barrier

**Figure 6:**
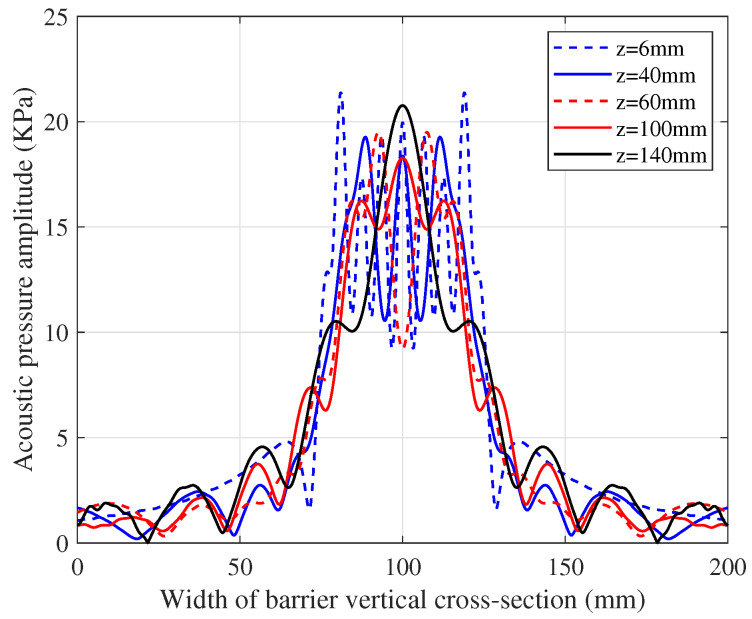
Line acoustic pressure distribution on the vertical cross-section of the barrier.

According to the COMSOL simulation, the plane acoustic pressure distribution function $p(x,y,z_{R})$ of the $P_{R}$ in Figure 3 can be obtained. Since the distance from the TX transducer is different, the value of function $p(x,y,z_{R})$ on the vertical cross-section of the metal barrier varies. To illustrate this phenomenon, five distances are taken to represent the distribution characteristics of $p(x,y,z_{R})$ as shown in Figure 6. In addition, it is known from Figure 6 that the side lobe of the power leakage has insignificant influence on the main lobe beam. The subsequent part of this paper would only consider the SEPAP distribution in the near-field.

**Figure 7:**
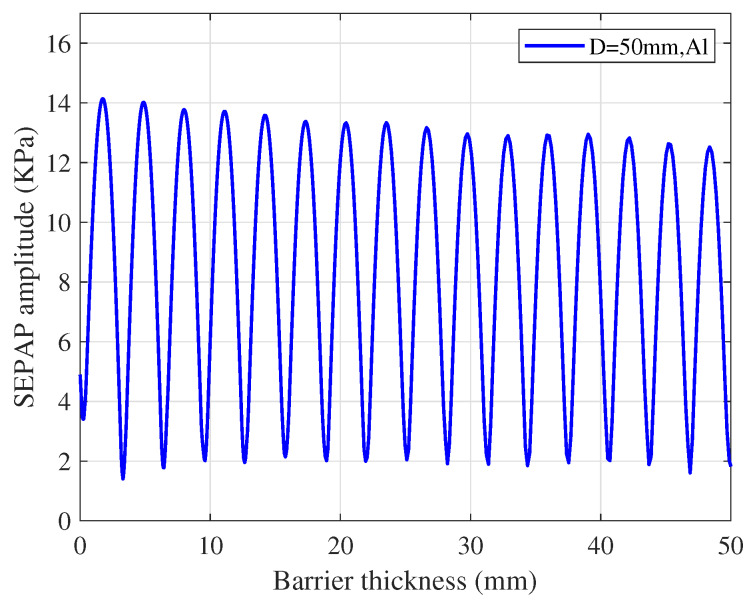
Amplitude variation of Spatial Equivalent Plane Acoustic Pressure (SEPAP) over the thickness the barrier.

According to Equation (7), the SEPAP for different distances can be obtained. The distribution of SEPAP in a metal aluminum barrier under the condition of transducer diameter D=50 mm is shown in Figure 7. We can find that the SEPAP amplitude varies approximately sinusoidally with the increase of the metal barrier thickness. In the near-field, the local maximum of SEPAP amplitude decreases as the barrier thickness increases. When the barrier thickness is an integral multiple of the half acoustic wave length, the SEPAP amplitude reaches the local maximum.

## 4. System Design

**Figure 8:**
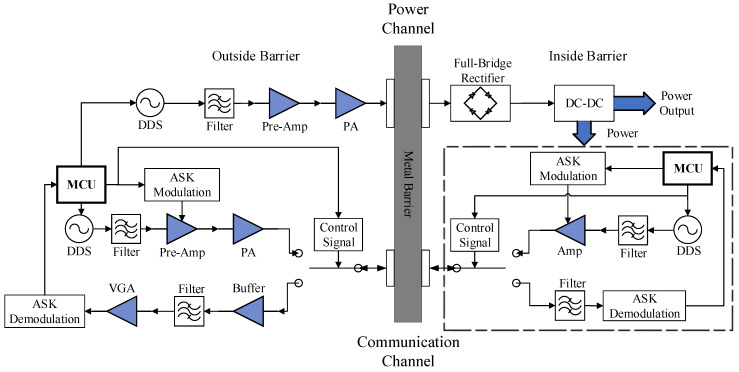
Block diagram of circuits for simultaneous transfer of power and data through metal.

In order to develop a low-cost, low-complexity and small-sized prototype for simultaneous transfer of power and data through metal, we propose a new system shown in Figure 8 for energy feed and bidirectional communication without using high-performance and high-cost devices.

We use two pairs of transducers for simultaneous transmission of energy and data respectively. Because the lower frequency can achieve deeper penetration [25] and the higher frequency provides broader bandwidth, we use PZT pairs with resonant frequencies of 1 MHz and 2 MHz for power transfer and communication, respectively. The subsequent sections will detail the implementation of each part.

### 4.1. Power Transfer Channel

In power transfer channel, the transmitting and receiving parts are designed respectively. At the transmitter side, a direct digital synthesizer (DDS) chip, AD9834, is used as the source for generating precise frequency 1MHz signal. Since the amplitude of the signal from AD9834 is low, we use OPA690 as the preamplifier and a high-voltage op-amp ADA4870 as the power amplifier to drive piezoelectric transducers. At the receiver side, the power for the system inside metal barrier is output from the received AC signal sequentially through a full-bridge rectifier and a DC-DC regulator. Due to the maximum output amplitude of ADA4870 is 40 $V_{PP}$, the rectifier is composed of 4 SS56 diodes, with the repetitive peak reverse voltage as 60 *V*. In order to improve the conversion efficiency and adapt to a wide voltage input range, we choose XL4005 as the DC-DC chip. The generated power is supplied to two parts. One part is the existing modules inside the sealing metal, and the other part is the communication circuits.

### 4.2. Bi-Directional Communication Channel

For through-metal communication, we use binary Amplitude Shift Keying (2ASK) scheme to simplify the modulation and demodulation system, and use Time Division Duplex (TDD) to achieve bi-directional communication. The signal link is divided into a forward link (from outside to inside) and a reverse link (from inside to outside). Since the central frequencies, i.e., 2 MHz, is very low and there is no silicon chips available for half-duplex switching. Since the amplitude of the signal leaked from the power channel may as high as 10 V, which is larger than the power supply rail of typical analog multiplexer. Thus, we choose relay as the Single-Pole Double-Throw (SPDT) rather than analog multiplexer here. To reduce power cost, the magnetic latching relay, which consumes power only in switching process, is chosen.

The signal generation outside metal barrier is similar to the power channel, while a 2ACK modulator is used to control the preamplifier. When the data bit is 1, the preamplifier is open. When the data bit is 0, the preamplifier is shutdown by the modulator. To lower the power consumption of the system inside metal barrier, we use OPA690 as an amplifier in reverse link instead of power amplifier. At the same time, a variable gain amplifier (VGA) is added to the signal receiving path outside the metal barrier to overcome the weak signal problem from the reverse link. The gain of VGA is determined by two factors. One is the different attenuation of the channel caused by different thickness of metal barrier. Another one is the output from the VGA must meet the input requirements of the 2ASK demodulation module. To isolate the interference from the power channel, a bandwidth filter has been added at the front-end of the receiving path.

## 5. Experiment Verification and Analysis

### 5.1. Experiment on the Power Transfer Channel

In this section, we use experiments to verify the relation between power transfer efficiency and SEPAP and explore the impact of transducer size on power transfer efficiency.

#### 5.1.1. Experimental Platform

**Figure 9:**
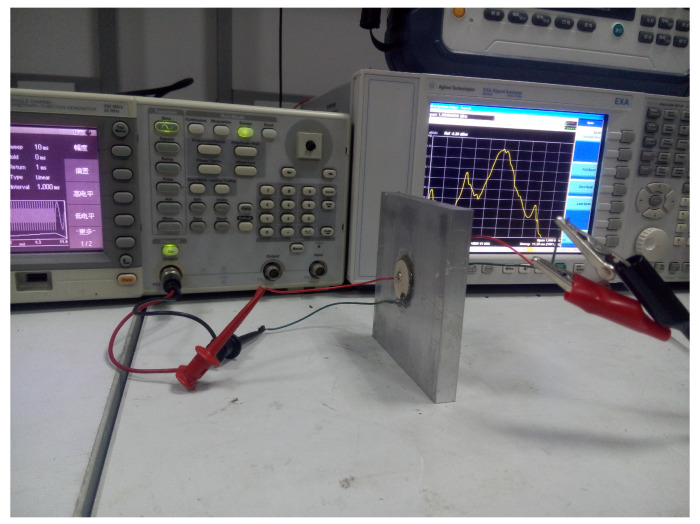
Experimental platform for power transfer.

We develop an experiment platform as shown in Figure 9. Details are given in the following. Metal aluminum plates with thickness from 1 mm to 25 mm with step 1 mm are used as the barriers. PZT4 transducers with diameters of D=50 mm, 40 mm and 25 mm are symmetrically coupled on both sides of the metal aluminum barriers through epoxy resin. A signal generator, Tektronix AFG3021B, is used to generate the loading sweep signal with frequency from 0.9 MHz to 1.2 MHz and amplitude of 10 $V_{PP}$ (25 dBm). The output signal on the receive transducer is connected to the Agilent N9010A spectrum analyzer for observation and measurement.

#### 5.1.2. Impact of Barrier Thickness on SEPAP and Power Transfer Efficiencies

**Figure 10:**
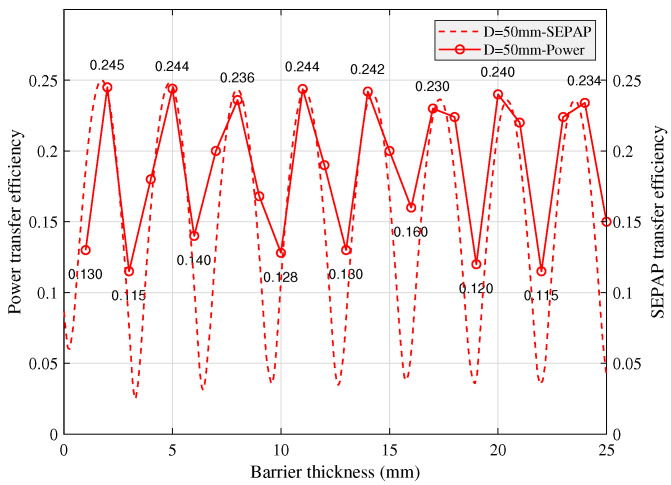
Relation between SEPAP and power transfer efficiencies over different barrier thickness.

The relation between SEPAP transfer efficiency and power transfer efficiency is shown in Figure 10. The power transfer efficiency varies approximately to the triangular wave and the local maximum decreases with the increase of the metal barrier thickness. Although the local maximum and minimum values of the power transfer efficiency and SEPAP transfer efficiency are not exactly matched with barrier thickness due to the low mechanical accuracy of epoxy resin coupling and metal barrier, but the trend of SEPAP fluctuation is generally consistent with the power transfer efficiency.

#### 5.1.3. Impact of Transducer Size on SEPAP and Power Transfer Efficiencies

**Figure 11:**
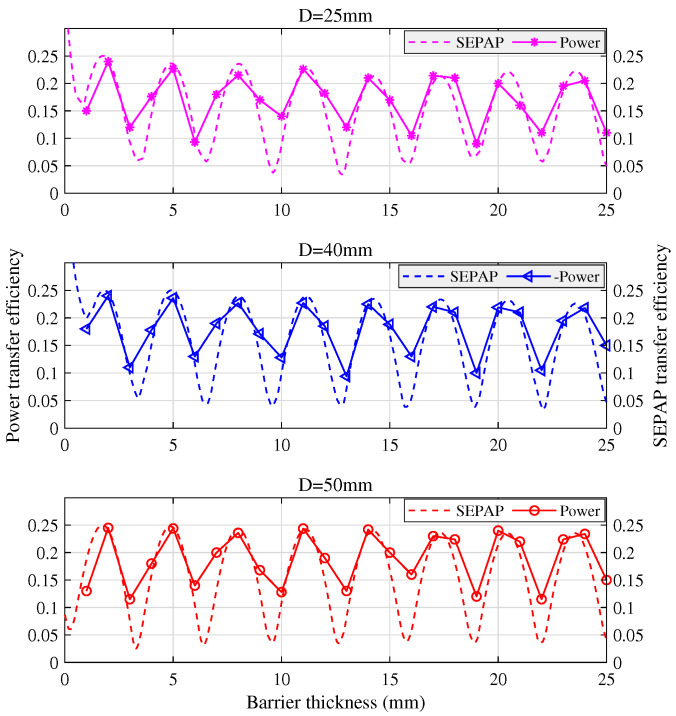
Impact of transducer size on SEPAP and power transfer efficiencies.

Both SEPAP transfer efficiency and the power transfer efficiency through an aluminum barrier by using transducer pairs with diameter D=50 mm, 40 mm, and 25 mm is shown in Figure 11. We can find that, with different transducer diameters, the variation tendency of SEPAP transfer efficiency and the power transmission efficiency is generally the same. The smaller the diameter of the transducer, the more rapidly the local maximum of power efficiency decreases with the barrier thickness.

#### 5.1.4. The Ratio between SEPAP and Power Transfer Efficiencies

**Figure 12:**
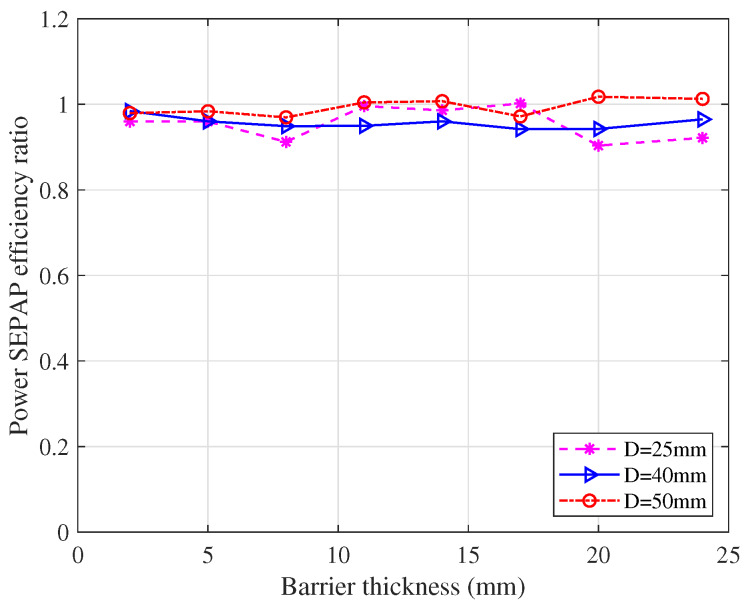
The ratio between SEPAP transfer efficiency and power transfer efficiency.

For further study of the relation between SEPAP and power transfer efficiencies through metal, we calculate the ratio between SEPAP local maxima and power local maxima and give it in Figure 12. From Figure 12, we can find that the ratio is almost fixed although there exists variation caused by the practical testing deviations. Thus, the impact of the diameter of the transducer on the ratio of local maxima SEPAP and power can be neglected. Therefore, we can use SEPAP transfer efficiency to indicate the power transfer efficiency through metal.

### 5.2. Experiment on the Communication Channel

#### 5.2.1. Experiment Platform

The experiment platform is similar to the test for the power channel. We use PZT with diameter of 50 mm and resonance frequency of 2 MHz. The barrier is an square aluminum metal baffle with side length of 200 mm and thickness of 20 mm, 30 mm, 40 mm, and 50 mm, respectively. The output of the receive transducer is connected to a 50 Ω load. Both the signal generator and the receive transducer are monitored by an oscilloscope through two synchronized channels.

#### 5.2.2. Time-Domain Response

**Figure 13:**
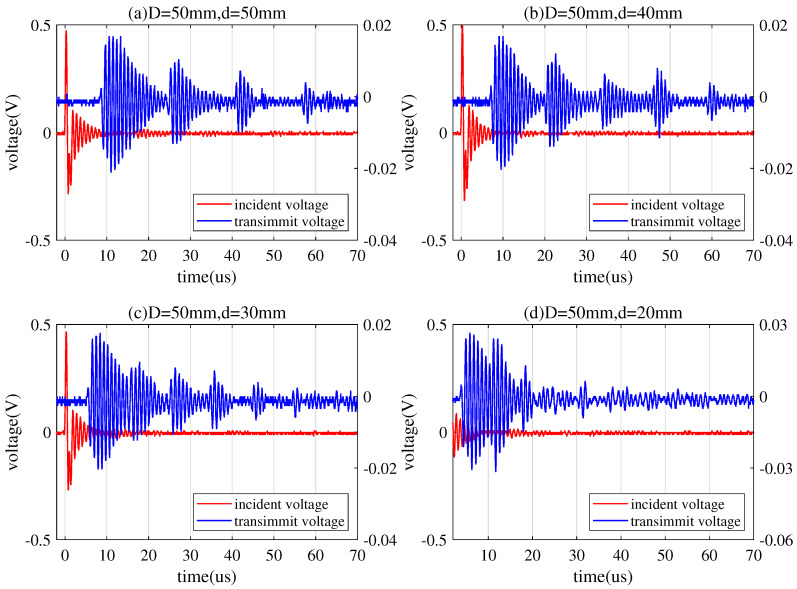
Pulse response with different barrier thickness.

In Figure 13, the red signal is the transmitted signal that is loaded into the transmitting transducer, and the blue signal is the received signal after passing through the channel. When a pulse signal is excited to the transmitting transducer, the signal received at the receiving transducer is ringing, i.e., the vibration decay of the impulse input. The formation of these trailing pulses (wave packets) is the elastic energy accumulated in the PZT during the input period of the electrical signal. During the period of no electric signal, the elastic energy in the PZT is continuously released [30]. These wave packets will attenuate with time. The larger diameter of the transducer, the more particles that simultaneously gain energy on the transducer, the slower energy releases.

In addition, there are multiple echoes in the received signal. The multiple echoes are generated due to the impedance mismatch between the transducer and the metal barrier. Thus, the acoustic waves will be reflected and transmitted at the interface of the transducer and the metal barrier. It can be seen from Figure 13 that the amplitude of the echo decreases with the number of reflections, and the attenuation loss decreases with the distance of the signal propagating in the metal barrier.

#### 5.2.3. Coherence Bandwidth Analysis

Multipath effects can cause severe inter symbol interference (ISI), which seriously degrades the communication rate. Next, we study the multi-path intensity distribution of the signal, and thus determine the maximum communication rate of the multi-path channel.

For a zero-mean and equivalent low-pass impulse response c(τ,t), its autocorrelation function is [31]

(8) $$R_{c}\left(\tau_{2},\tau_{1},\Delta t\right)=E\left[c^{*}\left(\tau_{1};t\right)c\left(\tau_{2};t+\Delta t\right)\right]$$

where $\tau_{1}$ and $\tau_{2}$ are uncorrelated delay, so Equation (8) can be written as

(9) $$E\left[c^{*}\left(\tau_{1};t\right)c\left(\tau_{2};t+\Delta t\right)\right]=R_{c}\left(\tau_{1},\Delta t\right)\Delta \left(\tau_{2}-\tau_{1}\right)$$

**Figure 14:**
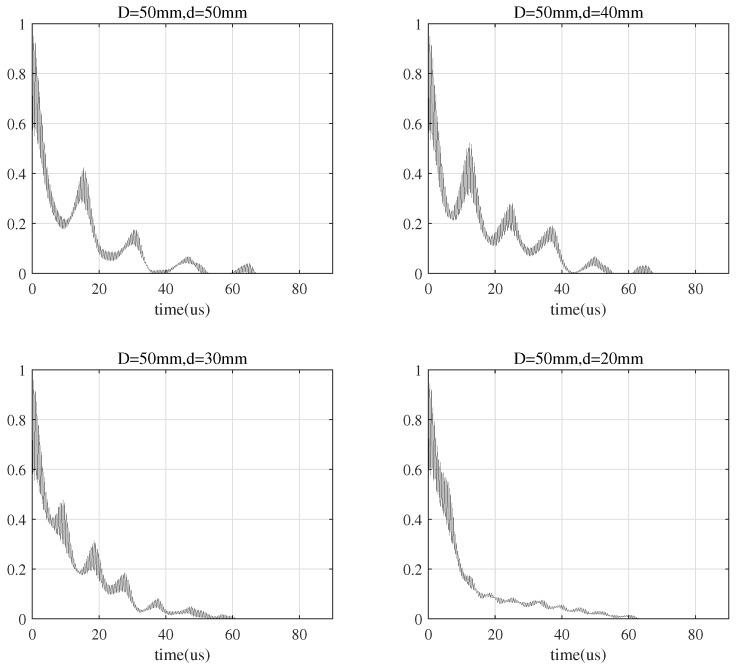
Multi-path intensity profile of the received signal by a transducer with diameter D=50 mm.

According to Equation (9), the multi-path intensity profile of the signal at the receiving end is shown in Figure 14. The multi-path intensity bulge that occurs during the decay process is related to the energy of multi-path echo signal. The number of local peaks is related to the diameter of the transducer, the smaller diameter of transducer, the less local peaks, i.e., the smaller the number of echoes. This is because that the strength of the generated signal by the transmitting PZT and the reflected signal from the receiving PZT is weaker when the diameter of the PZT is smaller. In addition, the multi-path delay is related to the diameter of the transducer, the larger diameter of the transducer, the longer multi-path delay it produces, thus the longer the coherence time. According to Figure 14, $T_{D=50}\approx 60$ is when d=20 mm.

Thus, the coherence bandwidth is approximately the reciprocal of the coherence time [31]

(10) $$F\approx \frac{1}{T}$$

We can obtain $F_{D=50}\approx 16.7$ KHz from Figure 14. If the bandwidth of the transmitted signal is larger than the coherence bandwidth, inter-symbol interference (ISI) will happen.

### 5.3. Systemic Experiment

**Figure 15:**
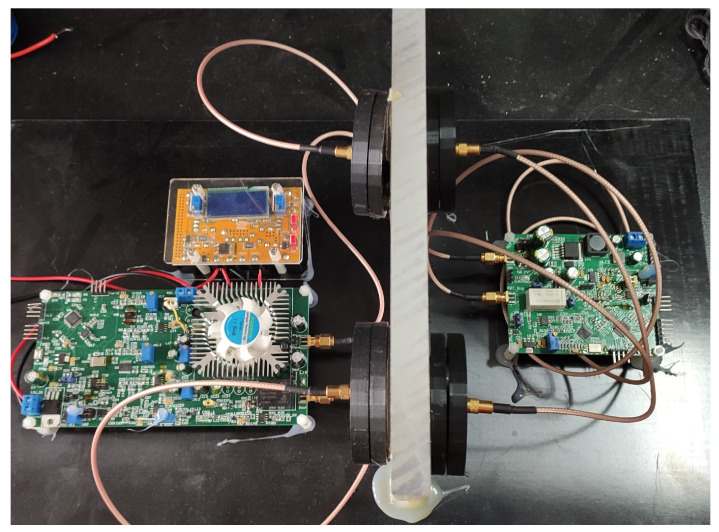
Prototype with two pairs of piezoelectric transducers (PZTs) for simultaneous power transfer and bi-directional communication.

We build up the prototype as shown in Figure 15. The final function and performance is tested through a 20 mm thick aluminum alloy plate. The maximum output power from the DC-DC module inside the barrier can reach 2.59 W (5 V/0.518 A), when the power consumption outside barrier is 12.72 W (12 V/1.06 A). This demonstrates that the system can achieve a power transmission efficiency of more than 20.3%.

**Figure 16:**
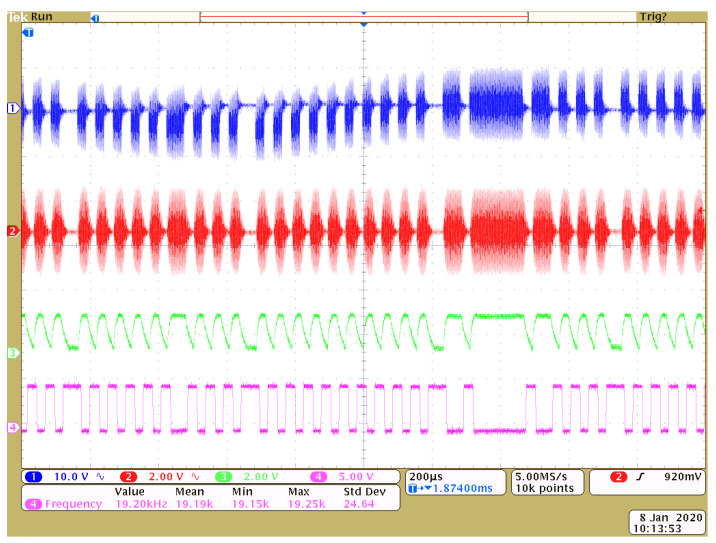
Practical waveforms on the transmitted ASK signal (blue), received Amplitude Shift Keying (ASK) signal (red), demodulated signal (green) and output digital signal (pink).

At the same time, the data communication rate higher than 38.4 Kbps can be achieved. This corresponds to the coherence bandwidth of 19.2 KHz, which is a bit larger than 16.7 KHz obtained in Section 5.2.3. This is because that the ASK demodulator can still decode signals with limited ISI. The transmitted ASK modulation signal (blue), received ASK modulation signal (red), demodulation (green) and output waveforms (purple) are shown from top to bottom in Figure 16. It proves that 38.4 Kbps can be achieved with a little ISI. Since all chips used in the prototype are cheap and the system design is easy, the system can be implemented easily in metal-sealing areas.

## 6. Conclusions

In this paper, we first propose to use the Spatial Equivalent Plane Acoustic Pressure (SEPAP) to characterize the complicated near-field acoustic distribution and then establish the through-metal power transfer system by using piezoelectric transducer. Based on SEPAP, the influence of the metal thickness and the transducer size on the equivalent pressure through metal is also studied by COMSOL.

We have designed a low-cost prototype by using two pairs of PZTs to achieve simultaneous transmission of energy and signals and tested the impact of PZT size and metal thickness on the performance. The match between PZT-based through-metal energy transfer efficiency and the SEPAP transfer efficiency has been explored. The achievable rate by using simple ASK modulation has also been analyzed. A practical test through a 20 mm aluminum alloy plate demonstrated that power transmission of 2.59 W with efficiency 20.3% and data communication rate up to 38.4 Kbps can be achieved simultaneously.

## Figures and Tables

**Table 1:** Material properties [25].

Category	Properties	Value
	Density (Kg/m${}^{3}$)	$7.5\times 10^{3}$
PZT4	Speed of acoustic (m/s)	$4.82\times 10^{3}$
	Acoustic impedance (Pas/m)	$3.615\times 10^{7}$
	Density (Kg/m${}^{3}$)	$2.7\times 10^{3}$
Aluminum	Speed of acoustic (m/s)	$6.42\times 10^{3}$
	Acoustic impedance (Pas/m)	$1.7334\times 10^{7}$
